# The footprint of gut microbiota in gallbladder cancer: a mechanistic review

**DOI:** 10.3389/fcimb.2024.1374238

**Published:** 2024-05-07

**Authors:** Shujie Liu, Weijian Li, Jun Chen, Maolan Li, Yajun Geng, Yingbin Liu, Wenguang Wu

**Affiliations:** ^1^ Joint Program of Nanchang University and Queen Mary University of London, Jiangxi Medical College of Nanchang University, Nanchang, Jiangxi, China; ^2^ Department of Biliary-Pancreatic Surgery, Renji Hospital, School of Medicine, Shanghai Jiao Tong University, Shanghai, China; ^3^ Shanghai Key Laboratory of Biliary Tract Disease Research, Shanghai, China; ^4^ State Key Laboratory of Oncogenes and Related Genes, Shanghai Cancer Institute, School of Medicine, Shanghai Jiao Tong University, Shanghai, China; ^5^ Shanghai Research Center of Biliary Tract Disease, Shanghai, China

**Keywords:** bacteria, gallbladder cancer, virulence factors, metabolites, chronic inflammation, biofilm formation, viruses, fungi

## Abstract

Gallbladder cancer (GBC) is the most common malignant tumor of the biliary system with the worst prognosis. Even after radical surgery, the majority of patients with GBC have difficulty achieving a clinical cure. The risk of tumor recurrence remains more than 65%, and the overall 5-year survival rate is less than 5%. The gut microbiota refers to a variety of microorganisms living in the human intestine, including bacteria, viruses and fungi, which profoundly affect the host state of general health, disease and even cancer. Over the past few decades, substantial evidence has supported that gut microbiota plays a critical role in promoting the progression of GBC. In this review, we summarize the functions, molecular mechanisms and recent advances of the intestinal microbiota in GBC. We focus on the driving role of bacteria in pivotal pathways, such as virulence factors, metabolites derived from intestinal bacteria, chronic inflammatory responses and ecological niche remodeling. Additionally, we emphasize the high level of correlation between viruses and fungi, especially EBV and *Candida* spp., with GBC. In general, this review not only provides a solid theoretical basis for the close relationship between gut microbiota and GBC but also highlights more potential research directions for further research in the future.

## 1 Introduction

Gallbladder cancer (GBC) is the most common malignant tumor of the biliary system, with the 6th highest incidence across malignant tumors of the gastrointestinal tract (Song et al., 2020a). According to Global Cancer Incidence, Mortality and Prevalence (GLOBOCAN) 2020 Statistics, there are more than 115,949 new cases and 84,695 new deaths of GBC per year (Sung et al., 2021). A systematic analysis showed a 76% and 65% increase in the incidence and mortality of GBC from 1990 to 2017, respectively (Ouyang et al., 2021). Its underlying causes are not fully understood, but cholelithiasis has been recognized as a major risk factor (Chen et al., 2022a), as confirmed by the fact that more than 51.1% of GBC incidence is associated with gallbladder stones (D’Afonseca et al., 2020). Other risk factors for GBC include senior age, female sex, infections, heredity, adenomatous polyps of the gallbladder, choledochal cysts, porcelainized gallbladder, anomalies of the biliopancreatic ductal junction, ulcerative colitis and metabolic disorders syndromes (Albaugh et al., 2019).

Due to the challenge of early diagnosis and the high malignant degree of late diagnosis, there is no specific and effective treatment for GBC. Similar to the vast majority of malignant tumors, surgical resection is still the only available curative strategy for GBC (Ying et al., 2021), but only 10 percent of patients are currently in a stage suitable for surgical resection (Goel et al., 2021). Incidental GBC cases are often found by pathological examination after cholecystectomy, requiring secondary surgery (Ethun et al., 2017), while symptomatic patients are usually at an advanced stage who are not suitable for surgery or have poor surgical outcomes (Liu et al., 2022b). Unfortunately, regardless of whether GBC is known or unknown preoperatively, the R0 resection rate remains suboptimal even with aggressive radical surgical resection (Benkhaled et al., 2023). Chemoradiotherapy is the preferred treatment for advanced GBC, but the lack of chemotherapy options and the insensitivity of radiotherapy lead to poor patient outcomes (Hakeem et al., 2019). Effective and safe targeted drugs and immunosuppressants for GBC have not yet been developed in recent years (Zuo et al., 2022; Wu et al., 2023). Therefore, most GBC patients have an extremely poor prognosis, with an overall 5-year survival rate of 5% (Wang et al., 2021a).

The human gut microbiota is an extremely diverse and complex ecosystem, which participates in various host physiological processes and contributes significantly to aspects of human health (Naseri et al., 2022). Although the commensal gut microbiota provides substantial benefits to the host, it is also a contributor to gallbladder injury under pathological conditions (Song et al., 2020b; Hu et al., 2023). Many studies confirmed that diverse gut microbes are involved in the development of GBC, which may potentially inspire the development of cancer therapies targeting gut microbes (Tsuchiya et al., 2018). In this review, we outline the latest research progress and summarize the roles and molecular mechanisms of gut microbiota in fostering GBC, focusing on bacterial driving effects in key pathways such as virulence factors, metabolites derived from intestinal bacteria, chronic inflammatory responses and ecological niche remodeling. Additionally, we highlight the high relevance of gut microbes, especially EBV and *Candida albicans (C. albicans)*, to GBC.

## 2 The gut microbiota in GBC progression

The human gut microbiota is composed of trillions microorganisms that live primarily in the digestive system, including bacteria, viruses, fungi, mycoplasmas and spirochetes (Kaewarsar et al., 2023). Among those, bacteria are the main inhabitants and based on natural properties, they are sorted into dozens of bacterial phyla, containing 500-1,000 species (Fujimori, 2021). Powerfully, the diversity of gut microbiota means a large number of genes and enzymes, which supports an enormous metabolic capacity and may be one of the most complex networks in human (Makarewicz et al., 2021). Undoubtably, the imbalances, dysfunctions and disorders of gut microbiota are acknowledged to be indicators of illness or adverse health status, such as metabolic syndrome, colorectal cancer and GBC (Hou et al., 2021).

Infection is a major factor in the development of approximately 16% of cancers (Simin et al., 2020), and the occurrence and progression of GBC are closely relevant to numerous gut microbiota in the tumor microenvironment. In this section, we provide a comprehensive overview on compositional alterations of gut microbiota in GBC patients, advancing the functional understanding of microbes involved in the progression of GBC. Gallbladder bile is the gold standard for biological fluid detection of GBC, because bile is secreted by the liver, stored by the gallbladder and exposed to the human gut microbiota through ducts (Keshavarz et al., 2022; Wheatley et al., 2022), while bioassay from gallstone, bile duct, gallbladder tissue or stool samples is also feasible. First, the research on *Salmonella* serotype Typhi (*S.* Typhi) infection in gallbladder tissue of genetically susceptible mice confirmed that *Salmonella* spp. is pathogenic and a causative bacterium of GBC (Scanu et al., 2015). Importantly, chronic *Salmonella* spp. carrier within bile, gallstones, tissue, or stool samples is associated with GBC, especially in areas with a high prevalence of typhoid fever (Koshiol et al., 2016). Second, *Helicobacter* infection has been shown to increase the risk of GBC, but the findings are inconsistent. *Helicobacter pylori* (*H. pylori)* in bile duct specimens may potentially be associated with GBC through the formation of bile duct stones (Cherif et al., 2019), and a clinicopathological study highlighted the role of *H. pylori* in exacerbating mucosal lesions of gallbladder, such as mucosal hyperplasia, metaplasia and lymphoid infiltration, which are considered potential precancerous lesions after infecting the gallbladder tissue of patients (Hassan et al., 2015). These results suggest a pathogenic role of *H. pylori* in inducing GBC in patients. However, meta-analysis and case-control studies showed that *Helicobacter bilis (H. bilis)* in the hepatobiliary ducts of gallstone and GBC samples does not increase the risk of gallbladder cancer. This study emphasizes that the increased risk of GBC observed in earlier studies may be indirectly due to gallstone factors, and not related to *H. bilis* infection. Unfortunately, in another study, *Helicobacter* spp. was not detected in bile of any GBC patients (Tsuchiya et al., 2018). Third, a bile metagenomics experiment verified that increases in specific bacterial taxa (*Leptospira, Mycoplasma gallisepticum*) and their functions are directly associated with lipid classes such as lysophosphatidyl inositol, ceramide 1-phosphate and lysophosphatidyl ethanolamine, initiating GBC progression (Sharma et al., 2022). The identified bacterial taxa and core bile lipid/metaproteome may provide universal utility for early diagnosis of GBC, but the causal relationship between these bacteria and GBC still needs to be further explored. In addition, by next-generation 16S rRNA sequencing, *Fusobacterium nucleatum* and *Escherichia coli (E. coli)* were found to be the dominant species in bile of GBC patients, but whether these bacteria are directly related to the development of GBC needs further study (Tsuchiya et al., 2018). According to the aforementioned findings, a variety of gut microbiota have obviously and significantly functional associations with GBC progression, and the underlying mechanisms are worth further aggregating and exploring.

Recently, the widespread application of next-generation sequencing technologies has defined unique microbiota characteristics in different cancers (Poore et al., 2020). Based on mucosal biopsy samples of chronic calculous cholecystitis (CCC) and GBC provided by 7 patients, we conducted a metagenomic shotgun study of the biliary microbiome along the CCC-GBC sequence and identified a total of 2543 microbial genes with significant differences (Song et al., 2020b). Both CCC and GBC groups were stable at *Firmicutes, Bacteroidetes, Actinobacteria* and *Proteobacteria* populations. However, the diversity of biliary microbiota was significantly increased in GBC group compared with CCC group, as evidenced by remarkable changes in up to 25 species. Among them, *Acinetobacter junii, Basidioascus, Crepidotus, Enterococcus faecium*, *Fusobacterium* and *Peptostreptococcus* were positively correlated with GBC. Further gene abundance comparison between the two groups revealed that the GBC group was enriched in NAD‐dependent protein deacetylase, deoxyribonuclease V and chorismate mutase. These functional proteins seem to be highly associated with severe inflammation and malignant GBC changes. Therefore, our study indicates that despite sharing stable and permanently dominant species, GBC patients exhibit distinct composition in biliary microbiota compared to CCC cases. This is the first evidence for the presence of an altered biliary microbiota in GBC samples, which may contribute remarkably to the GBC progression.

## 3 Bacteria in GBC pathogenesis

The human gut microbiota consists of approximately 10^14^ microorganisms and plays a crucial role in host function, especially in immunity and metabolism (Tao et al., 2022; Niu and Meng, 2023). Many studies demonstrated that the gut microbiota is closely interrelated with GBC progression, but most relative mechanistic studies have primarily focused on bacteria. In this section, we provide a systematic and comprehensive overview of the potential mechanisms driven by bacteria in GBC, focusing on their driving role in key pathways such as virulence factors, metabolites derived from intestinal bacteria, chronic inflammatory responses and ecological niche remodeling (**Table 1**).

**Table 1:** The possible GBC-associated intestinal bacteria and their mechanisms of action.

Bacteria	Study Model	Mechanism of action	Effector	Ref
*Acinetobacter* spp.	Human	Biofilm formation	Biofilm	(Tajeddin et al., 2016)
*C. scindens*	Mouse	Bile and intestinal bacteria-derived metabolites	LCA, DCA	(Marion et al., 2019)
*Desulfovibrionales*	Human and Mouse	Bile and intestinal bacteria-derived metabolites	Cholesterol metabolism, Bas, H2S, FXR, CYP7A1, Abcg5/G8	(Hu et al., 2022)
*Enterobacteriaceae* (*Escherichia* and *Klebsiella* genus)	Human	Chronic inflammatory response	–	(Choi et al., 2021)
*Enterococcus* spp.	Human	Biofilm formation	Biofilm	(Tajeddin et al., 2016)
*Eubacterium* sp. *C-25*	Human	Bile and intestinal bacteria-derived metabolites	DCA	(Song et al., 2021)
*E. coli*	Human	Virulence factor (genome instability)	CDT	(Razaghi et al., 2017)
	–	Virulence factor (genome instability)	R-Loops, Topoisomerase	(Brochu et al., 2023)
	–	Chronic inflammatory response	CNF1, Rho GTPases,	(Munro and Lemichez, 2005)
	Human	Biofilm formation	Biofilm	(Tajeddin et al., 2016)
*H. bilis*	–	Dietary and intestinal bacteria-derived metabolites	LPS, N-acetyltransferases	(Griffiths et al., 2021)
		Chronic inflammatory response	LPS, N-acetyltransferases,	(Griffiths et al., 2021)
*H. hepaticus*	Mouse	Virulence factor (genome instability)	CDT	(Suerbaum et al., 2003)
	–	Dietary and intestinal bacteria-derived metabolites	LPS, Peptidoglycan	(Falsafi and Mahboubi, 2013)
	–	Chronic inflammatory response	LPS, CDT	(Falsafi and Mahboubi, 2013)
*H. pylori*	–	Virulence factor (genome instability)	P53, DNA topoisomerase, DNA polymerase III, IS200, IS607, IF-3, RRF, 30S ribosomal protein S8	(Wang et al., 2021b)
	Human	Virulence factor (oxidative stress injury)	iNOS, ROS	(Zhou et al., 2013)
	Human	Chronic inflammatory response	IL-1β, TNF-α	(Mishra et al., 2013)
*H. pullorum*	–	Dietary and intestinal bacteria-derived metabolites	LPS, N-acetyltransferases, glucose or galactose derivative	(Griffiths et al., 2021)
		Chronic inflammatory response	LPS, N-acetyltransferases	
*K. pneumoniae*	Human	Biofilm formation	Biofilm	(Tajeddin et al., 2016)
*Leptospira* spp.	Human	Bile and intestinal bacteria-derived metabolites	Lipid metabolism	(Sharma et al., 2022)
*M. gallisepticum*	Human	Bile and intestinal bacteria-derived metabolites	Lipid metabolism	(Sharma et al., 2022)
*Odoribacteraceae*	Human	Bile and intestinal bacteria-derived metabolites	LCA	(Sato et al., 2021)
*P. aeruginosa*	Human	Biofilm formation	Biofilm	(Tajeddin et al., 2016)
*Salmonella* spp.	Mouse	Virulence factor (genome instability)	c-MYC, Sopb, SopB, SopE, SopE2, AKT pathway	(Scanu et al., 2015)
	Human	Bile and intestinal bacteria-derived metabolites	Lipid metabolism	(Sharma et al., 2022)
*S.* Typhi	Mouse	Chronic inflammatory response	Immunoglobulins, IL-4, Stat6, GATA3	(González et al., 2019)
	Human	Chronic inflammatory response	Vi antigen, O antigen	(SChadich et al., 2016; Shukla et al., 2021)
	–	Chronic inflammatory response	Caveolin 1, EXOC2, MAPK1, mTOR/TORC Pathway, Ras Pathway	(Khan and Bano, 2021)
	Human and Mouse	Virulence factor (oxidative stress injury)	EPS, ROS, NO	(Hahn and Gunn, 2020)
	Mouse	Virulence factor (genome instability)	TT	(Thakur et al., 2022)
	Mouse	Virulence factor (genome instability)	CDT	(Guidi et al., 2013)
	Mouse	Virulence factor (invasive enzyme attack)	Avra	(Lu et al., 2010)
*S.* Paratyphi A	Human	Virulence factor (genome instability)	CDT	(Sepe et al., 2020)
*S. bovis*	Human	Chronic inflammatory response	–	(O’Brien et al., 2014)

### 3.1 Intestinal bacteria and their virulence factors

**Figure 1:**
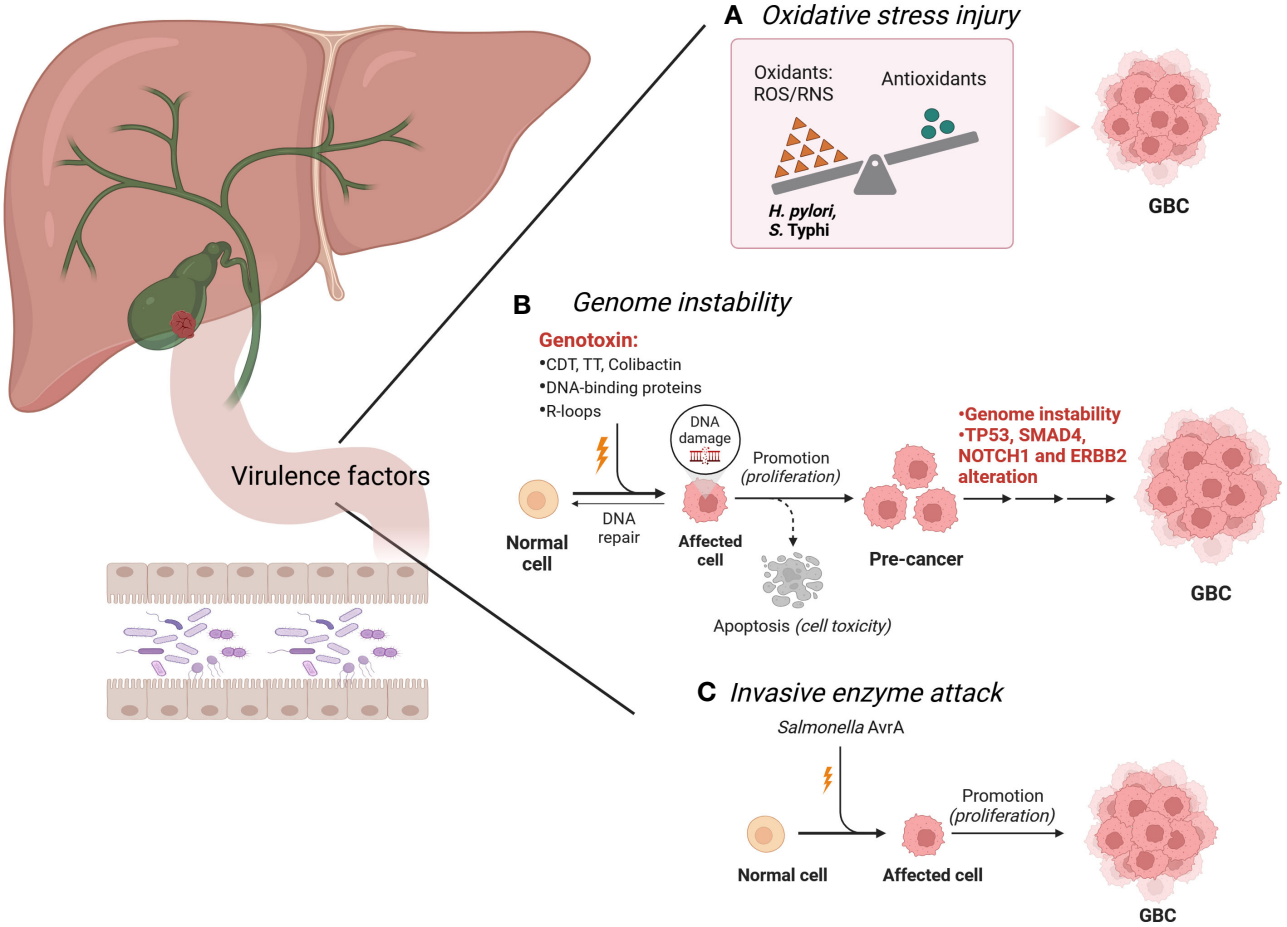
Intestinal bacteria and their virulence factors in the development of GBC. **(A)**. *H pylori* and *S.* Typhi produce high levels of ROS, which contribute to the precancerous lesions of the GBC. **(B)**. *H hepaticus*, *S.* Typhi, *Salmonella enterica, S.* Paratyphi A*, K pneumoniae, Enterobacteriaceae, H pylori* and *S. enterica* produce specific virulence factors, trigger the DNA damage response and genome instability, and eventually lead to GBC. These substances are also known as genotoxins, including CDT, TT, colibactin, DNA-binding proteins and R-loops. Genomic analysis showed that the TP53, SMAD4, NOTCH1 and ERBB2 mutations were found in the GBC cases. **(C)**. *Salmonella* spp. promotes GBC through invasive enzyme AvrA potentially. Image made with BioRender.com.

In patients with GBC, cancer cells are symbiotic with a large number of intestinal bacteria, such as *E. coli, H. hepaticus, H. pylori and Salmonella* spp. These bacteria have strong pathogenicity through the production of abundant virulence factors, which are mainly embodied by two aspects invasiveness and toxigenesis (López-Baena et al., 2019). Invasiveness is the capacity of pathogens to colonize, internalize, reproduce and spread in specific parts of the body (Nocera et al., 2023). Toxigenesis is the capacity to produce toxins that are categorized into exotoxins and endotoxins according to their sources, nature and roles (de Nies et al., 2021). Additionally, certain bacteria can produce invasive enzymes that harm body tissues and foster bacterial invasion and diffusion (Liu et al., 2019). Over the years, several bacteria are predisposing factors for the initiation and development of GBC. The damage of intestinal bacteria and their virulence factors to the host can be mediated via (i) genome instability, (ii) oxidative stress injury and (iii) invasive enzyme attack (**Figure 1**).

#### 3.1.1 Genome instability

As a heterogeneous disease, cancer is characterized by the presence of complex genomic instability, which is an extremely important hallmark of human cancer (Mayca Pozo et al., 2023). Genomic analysis showed that the number of mutations ranged from 1 to 15 in GBC cases, and a total of 171 mutations were identified. Among these, the most common alterations were found in TP53, SMAD4, NOTCH1 and ERBB2 (Mishra et al., 2022). Genomically unstable cancer cells can consistently generate new genetic variants, directly contributing to tumor progression (Weyburne and Bosco, 2021). Reliable experimental evidence supports that certain bacteria can mediate genome instability through specific toxins or proteins, which is closely associated with GBC carcinogenesis.

Certain bacteria can produce specific virulence factors, trigger the DNA damage response and genome instability, and eventually lead to disease (von Frieling et al., 2018). These substances are also known as genotoxins, including cytolethal distending toxin (CDT), typhoid toxin (TT) and colibactin (Wang and Li, 2022). CDT was the first described bacterial genotoxin and encodes three polypeptides (CdtA, CdtB, and CdtC) (Shenker et al., 2022). CDT induces double-strand breaks (DSBs), activates the ataxia telangiectasia mutated (ATM)-dependent DNA damage response and forms DNA repair complexes, triggering irreversible G2/M arrest of the cell cycle and apoptosis (Lara-Tejero and Galán, 2000; Zhang et al., 2018; Tatekawa et al., 2022). CdtB is the active constituent of CDT, which has functional homology with mammalian deoxyribonuclease I (DNase I) (Pons et al., 2021). It is noteworthy that *Helicobacter hepaticus (H. hepaticus)* (Suerbaum et al., 2003; Falsafi and Mahboubi, 2013), and *S.* Typhi (Guidi et al., 2013), and *Salmonella* serotype Paratyphi A (*S.* Paratyphi A) (Sepe et al., 2020) are bacteria that cause gene instability by producing CDT in patients with GBC. CDT also exists in *E. coli* (Wang and Fu, 2023), but whether *E. coli* promotes GBC through CDT needs to be further investigated. TT is a novel toxin found in *S.* Typhi and is composed of subunits CdtB, PltA and PltB (Du and Song, 2022). Interestingly, similar to the enzymatic subunit CdtB of CDT, TT can also mediate DNA damage and apoptosis by catalyzing CdtB, subsequently inducing typhoid-like symptoms (Thakur et al., 2022). Colibactin is also a common genotoxin that can cause double-stranded DNA breaks (Périchon et al., 2022). Colibactin is synthesized from the PKS genomic island of *Enterobacteriaceae* which consists mainly of *E. coli* and *Klebsiella pneumoniae (K. pneumoniae)* (Faïs et al., 2018), while *Enterobacteriaceae* are widely expressed in patients with GBC (Choi et al., 2021), implying the possibility that *Enterobacteriaceae (E. coli* and *K. pneumoniae)* induce GBC via colibactin. Therefore, mechanistic research on the *Enterobacteriaceae* genotoxin colibactin and its role in GBC is promising.

Bacteria can also produce multiple DNA-binding proteins, which interfere with the transmission of genetic information (including DNA replication, transcription and translation) and lead to genomic instability (Wang et al., 2021b). Typical representatives are *H. pylori* and *Salmonella* spp. *H. pylori* can inhibit the expression of p53 protein and normal cellular transformation by expressing multiple effectors targeting the host nucleus, which in turn leads to GBC progression (Arana and Kunkel, 2010; Wei et al., 2010). Its effectors include DNA topoisomerase, DNA polymerase III, bacterial insertion sequence 200 or 607 (IS200 or IS607), translation initiation factor-3 (IF-3), ribosome-recycling factor (RRF), 30S ribosomal protein S8 and various uncharacterized proteins (Wang et al., 2021b). DNA topoisomerase, DNA polymerase III, IS200 and IS607 cause genomic instability in infected cells by interfering with the process of gene replication (Bao and Jurka, 2013; Subramaniam et al., 2014). Among them, IS200 and IS607 encode a transposase (TnpA) and a protein (TnpB), respectively, thought to be methyltransferases (Wang et al., 2021b). Conversely, IF-3, RRF and 30S ribosomal protein S8 can disrupt protein synthesis by altering the transcription and translation processes of genes. In genetically predisposed mice characterized by *TP53* gene mutation and c-MYC amplification, *Salmonella* spp. induced malignant transformation of gallbladder-like cells, leading to GBC outcomes (Scanu et al., 2015). Mechanistically, effector proteins (SopB, SopE and SopE2) secreted by the type 3 secretion system (TTSS or T3SS) of *Salmonella* spp. during infection activate cellular protein kinases by the mitogen (MAPK) and AKT pathways, which not only promote the intracellular survival of the bacteria, but also initiate and maintain the transformation state of cancer cells (Kuijl et al., 2007; Scanu et al., 2015).

The bacterial product R-loops may also lead to genomic instability of host cells and induce GBC. During gene transcription, R-loops are a three-stranded nucleic acid structure composed of RNA : DNA hybrids and unpaired non-template single strands DNA (ssDNA), while RNA : DNA hybrids are formed due to the difficulty of separating newborn mRNA molecules from template DNA strands (Gowrishankar et al., 2013). Due to fragile ssDNA and blocked replication forks, abnormal accumulation of R-loops poses a major threat to genomic stability (Xu et al., 2023). Recent studies elucidated the specific pathways in which R-loops produced by *E. coli* trigger genomic instability and the regulatory mechanisms by which various topoisomerases participate in different steps (Brochu et al., 2023). Notably, human mitochondrial genome changes are related to GBC. Mitochondrial genome mutation analysis in patients with GBC, especially of the D-ring region, indicated a wide range of point mutations and polymorphisms (Maurya et al., 2013). In the future, researchers need to explore the specific bacteria that lead to the mutations of the mitochondrial D-ring sequence in patients with GBC.

#### 3.1.2 Oxidative stress injury

Oxidative stress (OS) is a state of imbalance between oxidation and antioxidation *in vivo*, which tends to cause oxidation and can be evaluated by the presence of reactive oxygen species (ROS) (Didziokaite et al., 2023). Excessive OS can cause oxidative damage to intracellular biomacromolecules and affect the fine regulation of important signaling pathways (Li et al., 2023). In recent years, the roles of *H. pylori* and *S.* Typhi in the development of GBC through OS damage are extensively investigated. *H. pylori* infection, whose potential mechanism may be related to higher ROS levels, was associated with precancerous lesions of the gallbladder mucosa, including septicemia and adenomyomatosis (Zhou et al., 2013). Recent studies have shown that excessive ROS generated in *H. pylori* infection induces direct degradation of STAMBPL1 by cullin 1-RING ubiquitin ligase and the 26S proteasome, while STAMBPL1 can lead to cell apoptosis by reducing the anti-apoptotic protein survivin (Chaithongyot and Naumann, 2022). Thus, *H. pylori* infection causes excessive OS and produces redundant ROS, which in turn downregulates the STAMBPL1 and contributes to the survival of GBC cells. However, in patients with gallbladder disease, chronic infection of *S.* Typhi utilizes a biofilm state to persist in the host, maintaining resistance to host responses, especially oxidative stress. *S.* Typhi can tolerate OS through a combination of an extracellular polymeric substance (EPS) barrier and catalase (Hahn and Gunn, 2020). In addition, *S.* Typhi could also significantly increase the expression of antioxidant enzymes (superoxide dismutase and catalase) during OS injury via the quorum sensing system, which induces the emergence of persistent bacterial cell populations, thereby promoting its own chronic persistent infection (Walawalkar et al., 2016).

#### 3.1.3 Invasive enzyme attack

Pathogenic bacteria can synthesize invasive enzymes that assist in their colonization, propagation and spread throughout the body. Their commonality is that they loosen the structures and enhance the permeability of the tissue structures but generally do not damage the body. *Salmonella* spp. is a potent carcinogen underlying GBC, which is closely related to the invasive enzyme AvrA. AvrA is a bacterial protein released by *Salmonella* spp. through TTSS (Pilonieta et al., 2014). One study created a mouse model of persistent *Salmonella typhimurium* infection *in vivo* and observed liver abscess and *Salmonella* spp. translocation in the gallbladder, emphasizing the importance of AvrA in bacterial invasion, bacterial translocation and chronic infection (Lu et al., 2010). Another study has confirmed cellular localization of AvrA produced by *Salmonella* spp. in precursor lesions of mouse gut and human colorectal tumors during inflammation (Lu et al., 2017). Therefore, the exploration of the underlying mechanism of AvrA secreted by *Salmonella* spp. in GBC with has potential prospects.

### 3.2 Metabolites derived from intestinal bacteria

Intestinal bacteria carry out complex metabolic activities in the gut, providing not only energy and nutrients for host growth but also active metabolites that affect human physiology (Rossi et al., 2020). Microbial metabolism is responsible for producing or modifying approximately 36% of the small molecules found in the human bloodstream (Chen et al., 2023). The vast range of metabolic substrates and metabolites associated with bacteria in GBC is of great significance. Exogenous foods or endogenous bile are transformed by bacteria to generate active metabolites that are either harmful or beneficial to humans, such as lipopolysaccharide (LPS), peptidoglycan, trimethylamine (TMA) and secondary bile acids (sBAs).

#### 3.2.1 Dietary and intestinal bacteria-derived metabolites

Nutrients in dietary are metabolized by intestinal bacteria to multiple active metabolites which play a significant role in GBC development. Early GBC cases are associated with poor diet, including eating too few whole-grain foods, vegetables (radishes, green peppers and sweet potatoes) and fruits (mangoes, oranges, melons and papayas) and consuming too much mustard oil, red meat (beef and lamb) and tea (Pandey and Shukla, 2002; La Vecchia et al., 2003; Mhatre et al., 2020; Nie et al., 2022).

LPS, peptidoglycan and TMA are the active metabolites produced by intestine bacteria through degrading down the nutrients contained in above-mentioned foods, which are closely related to GBC. First, LPS can be synthesized by a range of *Helicobacter* spp. through metabolizing dietary sugars, which is strongly associated with the development of GBC (Van Dyke et al., 2016; Griffiths et al., 2021). Recent studies discovered that *Helicobacter pullorum (H. pullorum)*, an emerging pathogen that may be associated with gallbladder diseases, expresses the key enzyme N-acetyltransferase (Griffiths et al., 2021). *H. pullorum* may metabolize carbohydrates in food by N-acetyltransferase to N-acetylated sugars, which in turn form the LPS of the cell wall. Specifically, the food substrates metabolized by *H. pullorum* are dTDP-3-amino-3,6-dideoxy-d-glucose (glucose derivative) or dTDP-3-amino-3,6-dideoxy-d-galactose (galactose derivative) (Griffiths et al., 2021). *H. bilis* and more than 50 species of non-pylori *Helicobacter* spp. (NPHS) also possessed genes encoding the enzyme N-acetyltransferase, which exhibited similar LPS biosynthesis functions, indicating that the bacterial metabolite LPS oncogenic pathway is common in GBC carcinogenesis (Griffiths et al., 2021). Second, peptidoglycan biosynthesis is another product of bacterial metabolism of carbohydrates (Galinier et al., 2023). *H. hepaticus* may be a human pathogen that causes GBC, and the pathogenesis is highly likely to be the proinflammatory response that is mediated by peptidoglycans and LPS on the cell wall (Falsafi and Mahboubi, 2013). Third, TMA is a conventional bacteria-derived metabolite, and the main sources of TMA are choline, phosphatidylcholine (lecithin) and L-carnitine in metabolized food (Panyod et al., 2023). TMA enters the circulation and is catalyzed in the liver by the key enzyme flavin-containing monooxygenase 3 (FMO3), which converts it to trimethylamine N-oxide (TMAO) (Chen et al., 2019). The TMA/FMO3/TMAO pathway regulates lipid metabolism in the body, and high TMAO levels predict high risk of future disease, including atherosclerosis and gallstones (Chen et al., 2019; Belli et al., 2023). Typical bacteria involved in the metabolism of TMA and TMAO in mammals encompass *Enterobacteriaceae* (mainly *E. coli* and *K. pneumoniae*) (Hoyles et al., 2018), *Deltaproteobacteria* (Yoshimoto et al., 2021) and *Clostridia* (Yoshimoto et al., 2021). However, the particular link between TMA and gut microbiota metabolism in GBC has not been investigated.

#### 3.2.2 Bile and intestinal bacteria-derived metabolites

The main organic components of bile are bile acids (BAs), cholesterol and phospholipids, and the composition and proportions of these components undergo significant changes under the actions of intestinal bacteria in GBC (Gubatan et al., 2023). Biochemical epidemiological analysis of GBC showed that the bile salts, phospholipids and cholesterol components of patients with GBC were significantly different from those of gallstone and non-gallstone patient controls (Strom et al., 1996). The particular composition and proportion of bile components in GBC patients may be attributed to the bile metabolism by intestinal bacteria.

**Figure 2:**
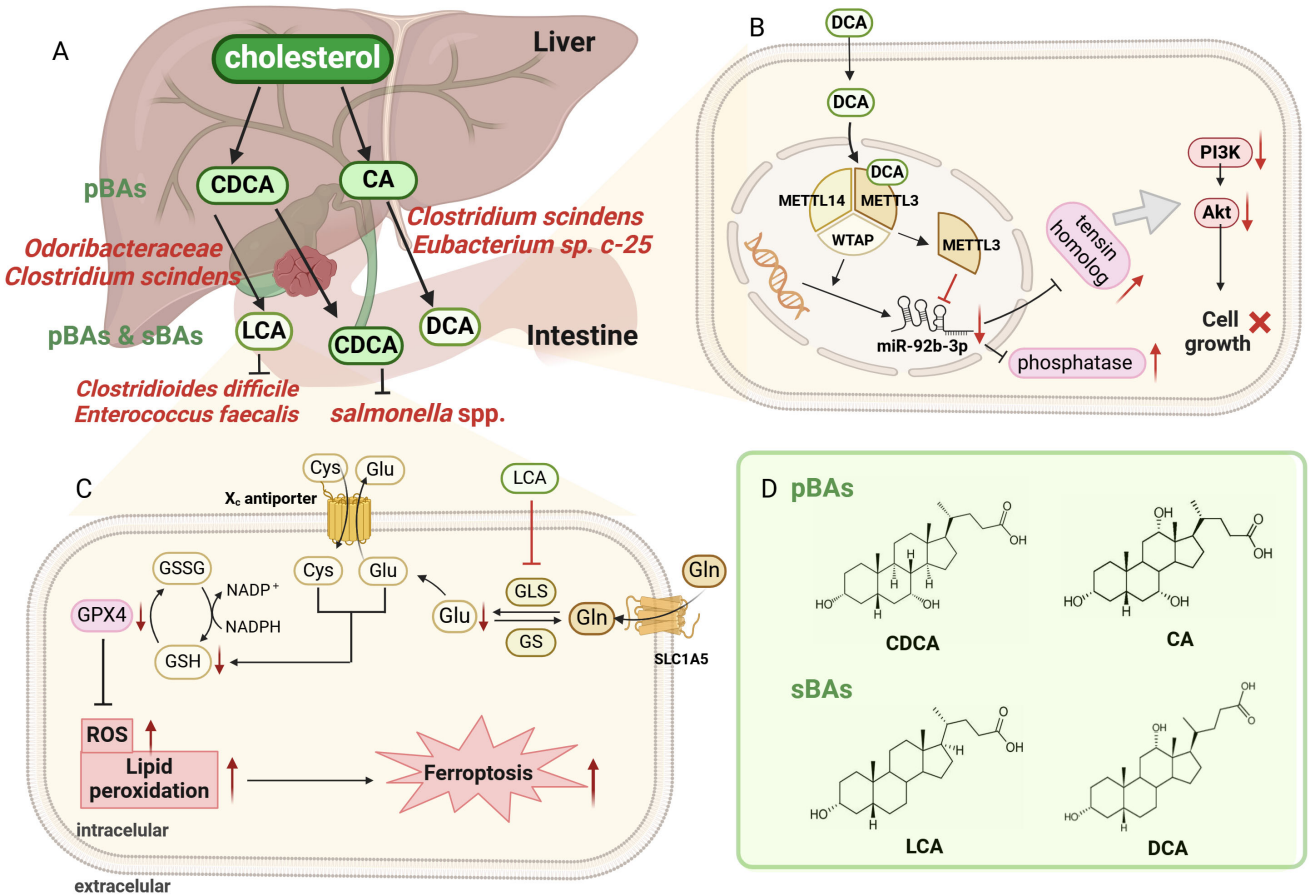
Overview of bile acids (BAs) metabolism by bacteria at intestine. **(A)**. The primary bile acids (pBAs) are directly synthesized by hepatocytes, including CDCA and CA, while the secondary bile acids (sBAs) undergo 7-dihydroxylation by bacteria, including LCA and DCA. *Odoribacteraceae and C scindens* metabolize CDCA to LCA, while *C scindens, Eubacterium* sp. *c-25* transform CA to DCA. Attractively, LCA can exert antibacterial effects on *Clostridioides difficile and Enterococcus faecalis*, reducing pathological infection and maintaining intestinal homeostasis. **(B)**. Schematic diagram of DCA-mediated cell death through dissociating METTL3–METTL14–WTAP complex. DCA facilitates METTL3 dissociation and downregulates miR-92b-3p expression, which subsequently increased the protein level (phosphatase and tensin homolog). These proteins inactivated the PI3K/AKT signaling pathway, eventually suppressing GBC tumor growth. **(C)**. Schematic illustration for LCA-mediated ferroptosis through inhibiting GLS. LCA reduced Glu synthesis by inhibiting GLS, leading to a rapid depletion of intracellular NADPH and GSH. Reduced GSH then restrains GPX4 level. but promoting ROS and lipid peroxidation, ultimately causing ferroptosis and cell death in GBC. **(D)**. The chemical structures of chenodeoxycholic acid (CDCA), cholic acid (CA), lithocholic acid (LCA) and deoxycholic acid (DCA). Image made with BioRender.com.

Most typically, bacteria actively metabolize cholesterol to various BAs at multiple locations (liver, gallbladder, intestine), with tremendous effects on host health (**Figure 2**) (Liu et al., 2022a). As the major organic constituents of bile, BAs are a collective term for several steroidal acids (Kiriyama and Nochi, 2021), and their microbial metabolism is a key point for GBC progression. In pathological conditions, an increase in the circulation rate of BAs not only increases the risk of developing GBC (Wu et al., 2020), but also results in a large number of derived metabolites, indirectly affecting key signaling pathways in GBC. BAs can be categorized into primary bile acids (pBAs) and secondary bile acids (sBAs) (Kolodziejczyk et al., 2019; Chen et al., 2022b). pBAs including chenodeoxycholic acid (CDCA) and cholic acid (CA) are synthesized directly by hepatocytes, whereas sBAs including lithocholic acid (LCA) and deoxycholic acid (DCA) undergo 7-dihydroxylation transformation by bacteria and enterohepatic recycling (Taylor and Green, 2018; Yang et al., 2022a). LCA, DCA and CDCA are confirmed to be intimately related to the development of GBC, acting as tumor suppressors in GBC. First, serum LCA is significantly decreased in patients with GBC, which is associated with poor clinical outcomes (Li et al., 2022). The anti-cancer mechanism of LCA in GBC can be explained from two aspects. On the one hand, LCA induces cellular ferroptosis and inhibits GBC cell proliferation by downregulating glutaminase-mediated glutamine metabolism (**Figure 2A**) (Li et al., 2022). On the other hand, LCA can also produce effective antibacterial effects against Gram-positive multidrug-resistant pathogens, including *Clostridioides difficile* and *Enterococcus faecalis*, which in turn mediates anti-cancer effect (Sato et al., 2021). This is demonstrated by long-lived centenarians with a unique gut microbiota, particularly rich *Odoribacteraceae* strains that produce LCA both *in vitro* and *in vivo*, facilitating the reduction of pathologic infections and the maintenance of intestinal homeostasis (Sato et al., 2021). Second, DCA is also significantly decreased in patients with GBC, which is associated with adverse clinical outcomes (Lin et al., 2020). Mechanistic studies confirmed that DCA can regulate GBC progression through N6-methyladenosine-dependent microRNA maturation (**Figure 2B**) (Lin et al., 2020). Specifically, DCA reduces miR-92b-3p expression by promoting the dissociation of METTL3 from the METTL3-METTL14-WTAP complex. The downregulation of miR-92b-3p promotes increased protein levels of target phosphatase and tensin homologue, which in turn inactivate the PI3K/AKT signaling pathway and inhibited tumor growth in GBC. *Clostridium scindens (C. Scindens)* can bioconvert BAs to LCA and DCA *in vitro* and *in vivo* (Marion et al., 2019), and *Eubacterium* sp. *c-25* is a producer of atypical DCA in humans (Song et al., 2021). Third, CDCA can bind and inactivate *Salmonella*, but there are currently no reports investigating specific microorganisms that metabolize CDCA in humans (Yang et al., 2023). The mechanism by which CDCA inactivates *Salmonella* spp. is directly binding and inhibiting the protein HilD, which is an important transcriptional regulator of bacterial virulence and pathogenesis.

There seems to be a close relationship between gut microbiota and cholesterol metabolism in GBC. *Desulfovibrionales* is closely related to gallstone and GBC by affecting cholesterol and BAs metabolism in the bile (Hu et al., 2022). *Desulfovibrionales* promotes intestinal cholesterol absorption by increasing the sBAs generation and BAs hydrophobicity, and conversely improves bile cholesterol secretion by inducing the expression of cholesterol transporter Abcg5/g8 in the liver. Interestingly, transplanting the microbiota by feces of gallstone patients into mice without gallstones eventually induced gallstone formation, verifying the important role of *Desulfovibrionales* in gallstone formation (Hu et al., 2022). Gallstones are the main risk factor for GBC, which are confirmed by a mouse model of gallbladder precancerous development (Rosa et al., 2020). In this model, a high cholesterol diet induces the early formation of gallstones, extensive inflammatory changes and even dysplastic lesions at advanced stages. Therefore, it is important to elucidate the role of *Desulfovibrionales* in cholesterol metabolism in GBC.

In GBC, lipid metabolism by intestinal bacteria can provide cancer cells with energy, biofilm components and necessary signaling molecules for sustainable proliferation, invasion and metastasis (Zhan et al., 2022). Bile multiomics analysis classified lipid species and microbial peptides and found that bile from GBC patients showed significant alterations in the lipidome and microbiota (Sharma et al., 2022). This was shown by a decrease in lipid classes (lysophosphatidylinositol, ceramide 1-phosphate, lysophosphatidylethanolamine), as well as an increase in bacterial taxa (*Leptospira*, *Salmonella* spp., *Mycoplasma gallisepticum*). This study confirmed that those bacteria and their enhanced function in metabolizing aforementioned lipids are closely related to GBC, which may provide feasibility for the early diagnosis of GBC.

### 3.3 Chronic inflammatory response

The immune response is an essential self-defense mechanism that protects the body from infection (Di Cara et al., 2019). Appropriate immune responses eliminate pathogens that are detrimental to organisms, but prolonged immune responses result in chronic inflammation, local tissue damage and even cancer (Bucciantini et al., 2021). Analyzing the relationship between 13 personal comorbidities and the risk of subsequent GBC, a recent study indicated that infection is a major risk factor for GBC, while local chronic inflammation and associated immune disorders are carcinogenic triggers (Hemminki et al., 2023), regardless of whether its origin is lithological or not (Cai et al., 2023). There may be multiple carcinogenic mechanisms involved, with common characteristics in changed signaling pathways and increased inflammatory factors (Rubini et al., 2018). Typical bacteria involved in this process are *S.* Typhi*, Helicobacter* spp.*, E. coli, Enterobacteriaceae* and *Odoribacteraceae*.

**Figure 3:**
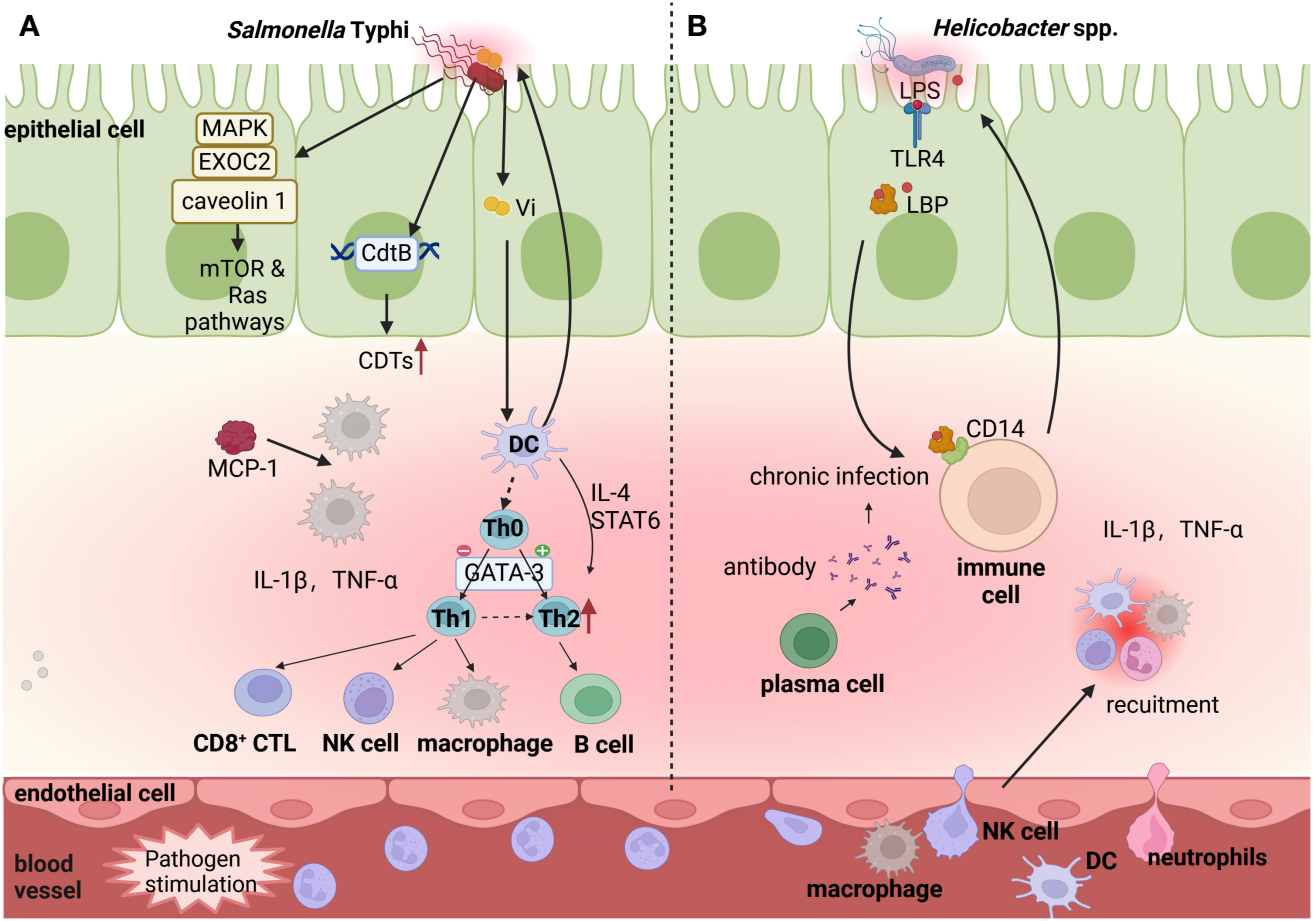
Potential function of bacteria-induced chronic inflammation in GBC progression. Commensal microbiota in the gut and gallbladder lumen stimulate innate and adaptive immune cells (DCs, Macrophages B cell and T cell), and release the inflammatory factors (IL-1β,TNF-α, etc.). **(A)**. *S.* Typhi increases levels of immunoglobulins, by activating Th2 upstream regulators (IL-4 and STAT6), Th2 major transcriptional regulator (GATA3) and elevating levels of T- and B-cells. Additionally, *S.* Typhi encodes CdtB to produce the toxic molecule CDTs and its Vi antigens recruit DCs and Macrophages, which promote the persistence of infection. *Salmonella* spp. interacts with several human proteins (caveolin 1, EXOC2, and MAPK), which can further regulate the human mTOR/TORC and Ras signaling pathways in inflammatory reaction. **(B)**. Some *Helicobacter* spp. releases surface LPS that transports to the surface of immune cells by lipopolysaccharide-binding protein (LBP) and binds to membrane protein CD14, regulating inflammation and immunity reaction. Image made with BioRender.com.

As a classic human-restricted pathogen of GBC, chronic *S.* Typhi infection is associated with gallbladder inflammation, which is strongly associated with GBC (**Figure 3**). Supporting it, *S.* Typhi was present in 40% of patients with GBC compared with 8% of total patients (Wernberg and Lucarelli, 2014). *Salmonella* spp. infection in the gallbladder triggers an intense inflammatory response, manifested by elevated levels (more than 10-fold) of proinflammatory mediators such as tumor necrosis factor-α (TNF-α), interleukin-6 (IL-6) and monocyte chemotactic protein-1 (MCP1) (Menendez et al., 2009). This may be due to the following mechanisms. First, by screening host-pathogen interactions and GBC targets through databases and performing functional overrepresentation analysis, study identified that *Salmonella* spp. interacted with several human proteins such as MAPK, RAC1, caveolin 1 and EXOC2 (Khan and Bano, 2021), inducing gallbladder adenocarcinoma which accounts for 85% of GBC. These proteins can regulate the various signaling pathways, incorporating PI3K/AKT/mTOR, Ras/Raf/MEK/ERK, MAPK/ERK, CREB/SP-1 and BSG, leading to GBC (Khan and Bano, 2021). Second, *S.* Typhi, the enterica serotype of *Salmonella* spp. that encodes CdtB, produces the toxic molecule CDTs (Lai et al., 2021). Due to immunomodulatory activity, CDTs may promote the persistence of infection, thereby prompting GBC (Mannion et al., 2022). Third, some studies determined the correlation between Vi antigen of *S.* Typhi in serum samples and GBC (Nath et al., 2008; Shukla et al., 2021). Importantly, Vi antigens on some *S.* Typhi capsules can regulate different proinflammatory signaling pathways in infected macrophages and dendritic cells, facilitating disease progression (SChadich et al., 2016). Therefore, it is necessary to explore how Vi antigens of *S.* Typhi promote the progression of GBC through the inflammatory pathway. Fourth, the establishment of chronic *S.* Typhi *i*nfection carriage model in mice with gallstones displayed a shift from an early Th1 proinflammatory response on day 7 to a later or chronic Th2 anti-inflammatory response on day 21 in gallbladder, which was characterized by increased levels of immunoglobulins, Th2 upstream regulators (IL-4 and STAT6), Th2 major transcriptional regulator (GATA3) and elevated levels of T- and B-cells (González et al., 2019). The underlying mechanism is that the biofilm state allows *Salmonella* spp. to resist the initial onslaught of the Th1 inflammatory response, while yet undefined events affect the shift of host immunity toward a more permissive type 2 response, explaining the persistence of *Salmonella* spp. and chronic infection in gallbladder disease. In conclusion, *S.* Typhi induces a chronic inflammatory response, which promotes the occurrence and development of GBC by altering various signaling pathways and releasing inflammatory mediators.

Multiple *Helicobacter* spp., including *H. pylori, H. hepaticus, H. pullorum* and *H. bilis* can also induce chronic inflammation, contributing to the development of GBC (**Figure 3**). Polymerase chain reaction showed that *H. pylori* exists in 54% of GBC samples, and the levels of inflammatory cytokines, such as IL-1β and TNF-α, are significantly elevated in *H. pylori*-positive samples (Mishra et al., 2013). As the known pro-inflammatory cytokines inducing gastric cancer, IL-1β and TNF-α mediate *H. pylori* to initiate a large number of neutrophils and lymphoid cells to infiltrate gastric mucosa, highly polarized to Th1 cytokine response, leading to gastric mucosal injury and disease (Mishra et al., 2013). However, more studies are needed to reveal the underlying mechanisms of IL-1β and TNF-α in the occurrence of GBC. Moreover, *H. hepaticus* is a human pathogen responsible for GBC, which is highly likely to induce a proinflammatory response through CDT toxins (Falsafi and Mahboubi, 2013; Pons et al., 2021). Long-term exposure to CDT of HeLa cells can promote furious cGAS-dependent type I interferon responses and modulate host immune responses (Pons et al., 2021). Therefore, *H. hepaticus* may be related with GBC carcinogenesis through CDT-mediated cellular immunity, but specific studies are still needed to determine. Additionally, *H. pylori, H. hepaticus, H. pullorum* and *H. bilis* can induce an inflammatory response in body via LPS (Griffiths et al., 2021), while LPS pathway proteins, containing lipopolysaccharide-binding protein (LBP) and soluble CD14 (sCD14), are powerfully associated with GBC (Van Dyke et al., 2016). The basic mechanism is that bacteria invade body and release surface LPS, which is then transported to the surface of immune cells by LBP and then binds to membrane protein sCD14, playing a regulatory role in inflammation and immunity (Yang et al., 2021; Chen and Lin, 2022). A comparative study in Shanghai, China examined the relationship between LPS, LPS pathway proteins (LBP and sCD14) and GBC by logistic regression, and its results indicated that both LBP and sCD14 were positively correlated with 63 inflammation-related markers, and were associated with GBC in adults, suggesting that LPS pathway proteins are associated with adult GBC by mediating systemic inflammation. Despite the fact that there have been relatively few studies specifically examining the relationship and mechanism between the LPS pathway and GBC, the broader literature on colorectal cancer (Chen et al., 2011) and gastric cancer (Li et al., 2019) reinforces the role of bacteria in cancer development by mediating inflammation via LPS pathway signaling, supporting that LPS pathway may contribute to gallbladder carcinogenesis through inflammatory process.

The existence of several bacteria in GBC has been identified, containing *E. coli, Enterobacteriaceae and Streptococcus bovis (S. bovis)*, but their definitive roles remain to be elucidated. First, *E. coli* possesses high pathogenicity due to the virulence factor cytotoxic necrotizing factor 1 (CNF1), resulting in a sustained inflammatory response and a high risk of cancer. On the one hand, CNF1 produced by *E. coli* induces the activation and maturation of human monocyte-derived dendritic cells, as indicated by the increased ability to secrete inflammatory cytokine and stimulate the proliferation of allogeneic naive CD4+ T cells, which play a role in colon tumor of mice (Gall-Mas et al., 2018; Chat et al., 2023). On the other hand, CNF1 can activate Rho GTPases via deamidation, which then accompanied by Rho-induced ubiquitin-mediated proteasomal degradation (Carlini et al., 2021). The activation and degradation of Rho reduces the threshold of the cellular inflammatory response and triggers the cellular inflammation in various cancers (Chat et al., 2023). Considering that *E. coli* is the most commonly isolated strain from the bile of GBC patients and the promoting role of CNF1 in a variety of cancers, further studies are needed to elucidate the exact role of CNF1 produced by *E. coli* in GBC. Second, *Enterobacteriaceae* infections may produce a chronic inflammation, prompting GBC. Metagenomic sequencing of bile samples revealed a significant increase in the abundance of the *Enterobacteriaceae* family (including the *Escherichia* and *Klebsiella* genus) in GBC samples (Choi et al., 2021). Moreover, a progressive and notable increase in *Klebsiella* spp. is observed in the order of normal gallbladder, chronic cholecystitis and GBC. *Enterobacteriaceae* is crucial for the acute inflammatory response in the small intestines of mice by regulating the production of corticosterone, but its specific mechanism in GBC inflammation still needs to clarify by further research (Menezes-Garcia et al., 2020). Third, *S. bovis* infection is associated with GBC. A case report showed that a 77-year-old man presented with *S. bovis* bacteremia during acute cholecystitis, and then found a concurrent GBC during cholecystectomy. This is the first reported case of *S. bovis* infection and coexisting GBC (O’Brien et al., 2014). *S. bovis* infection not only advances colorectal cancer by recruiting CD11b^+^TLR-4^+^ cells and releasing inflammatory cytokines (IL-6, IL-1ß and TNF) (Deng et al., 2020), but also boosts gastric cancer by affecting immune cells (CD3+ T cells and NK cells) in the peripheral blood (Qi et al., 2019; Zi et al., 2022). These three bacteria are closely connected to GBC by chronic inflammatory response, but the exact pathogenic links have not been determined.

### 3.4 Biofilm formation

**Figure 4:**
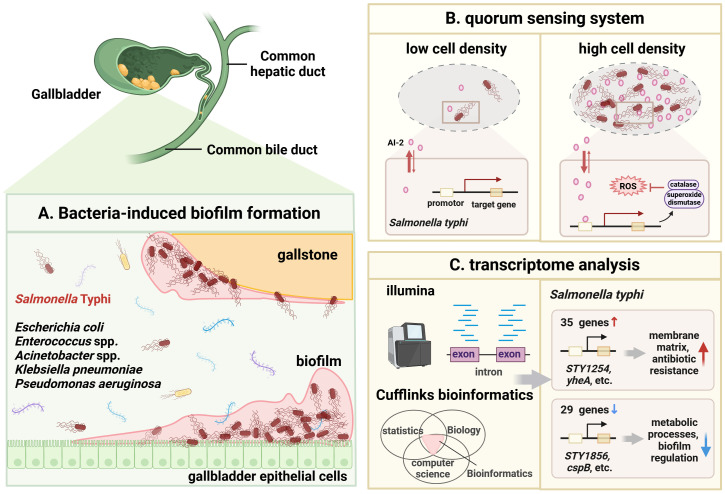
Possible role of biofilm-producing bacteria in GBC development. **(A)**. Schematic diagram of bacteria-induced biofilm formation. The gallstone and gallbladder epithelial cell are the ecological niche for *S.* Typhi to attach and form biofilm. Chronic infection with biofilm-forming bacteria *(Pseudomonas aeruginosa, E coli, K pneumoniae, Enterococcus* spp. and *Acinetobacter* spp.*)* also are found in GBC. Once biofilm is formed, bacteria can persist in the gallbladder and release carcinogenic molecules, thereby inducing GBC. **(B)**. Schematic illustration of quorum sensing (QS) systems in *S.* Typhi. At low bacterial density, the autoinducer (AI)-2 concentration is low. At high bacterial density, the AI-2 concentration reaches a threshold to induce corresponding gene expression to initiate QS system. The expressed catalase and superoxide dismutase help bacteria resist ROS and oxidative stress, adapt to the difficult bile environment and evade the host immune response. **(C)**. Transcriptomic analysis showed that the biofilm phenotype of *S.* Typhi allows bacteria to upregulate the expression of 35 genes (*STY1254, STY1255, yheA*, etc.) related to the membrane matrix and antibiotic resistance, downregulated 29 genes (*STY1856, yiiU, cspB*, etc.) related to metabolic processes and biofilm regulation. Image made with BioRender.com.

Due to the existence of biofilm, *S.* Typhi persistently exists in the gallbladder of patients with gallbladder disease (**Figure 4**). Biofilm is considered tightly associated with the pathogenicity and persistence of *S.* Typhi (González et al., 2022). On the one hand, biofilm formation is partially dependent on the quorum sensing (QS) system and persistent cell populations in gallbladder (**Figure 4B**) (Walawalkar et al., 2016; Liao et al., 2019). As a microbicide, bile leads to bacterial oxidative stress and the ROS production in the gallbladder. *S.* Typhi can remarkably increases the levels of antioxidant enzymes, namely catalase and superoxide dismutase, through the QS signal autoinducer-2 (AI-2), adapting to the harsh bile environment (Wattanavanitchakorn et al., 2014). When exposed to bile and treated with transient antibiotics, *S.* Typhi also formed persistent cell population with slow or arrested growth and significantly increase by up to 3-fold. The QS system and persistent cell populations may protect bacteria from bile environmental pressure and host immune response, maintain persistent bacterial infection and promote the development of GBC (Di Domenico et al., 2017). On the other hand, transcriptomic mechanism studies showed that the biofilm phenotype of *S.* Typhi allows bacteria to upregulate the expression of 35 genes related to the membrane matrix and antibiotic resistance (*STY1254, STY1255, yheA*, etc.), downregulated 29 genes related to metabolic processes and biofilm regulation (*STY1856, yiiU, cspB*, etc.), and enter a state of energy conservation in response to the stressful environment (Chin et al., 2017). Interestingly, gallstones and gallbladder epithelial cells are the ecological niche for *Salmonella* spp. to attach and form biofilm (**Figure 4C**) (Menendez et al., 2009). In addition, a study on the associations between different bacterial communities and biliary diseases in human bile samples confirmed the coexistence of different combinations of biofilm-forming bacteria (*Pseudomonas aeruginosa, E. coli, K. pneumoniae, Enterococcus* spp. and *Acinetobacter* spp.) (Tajeddin et al., 2016). The ability to form biofilm appears to be a need for multiple microbial infections in the gallbladder and biliary tract, but the mechanisms by which biofilm-forming bacteria aside from *S.* Typhi contribute to the progression of GBC need to be further explored.

## 4 Viruses in GBC pathogenesis

Viruses, especially Epstein-Barr virus (EBV) hidden in the human intestine, are also an important part of the gut microbiota that can promote the occurrence and development of GBC (Xiong et al., 2023; Zhang et al., 2023). Although viruses are extremely minuscule, they are flexible and promote tumorigenesis through different pathways involving signal transduction changes, the DNA damage response and immune regulation (Krump and You, 2018). Currently, based on IARC (International Agency for Research in Cancer) data, there are 7 commonly affirmative viruses that can cause cancer, including EBV, Hepatitis B virus (HBV), Hepatitis C virus (HCV), Kaposi sarcoma herpesvirus (KSV), human immunodeficiency virus-1 (HIV), human papilloma viruses (HPV) and human T-cell lymphotropic virus type 1 (HTLV) (Zapatka et al., 2020). Among these viruses, the first identified oncogenic EBV is implicated in a variety of cancers, including nasopharyngeal carcinoma, gastric cancer, lymphoma and GBC (Tong et al., 2022; Zhu et al., 2022).

Several studies revealed a close correlation between EBV and GBC. First, EBV infection usually has a long incubation period, and only when immune function is compromised does the host develop clinical symptoms and GBC. A 10-year-old HIV-positive female patient required emergency cholecystectomy due to the discovery of an empyema gallbladder, and subsequent gallbladder histopathology confirmed the presence of EBV-associated smooth muscle tumors involving the gallbladder (Mahlobo et al., 2012). Second, primary EBV infection can cause acute acalculous cholecystitis (AAC), especially in young women (Agergaard and Larsen, 2015). A literature search identified 26 cases of acute EBV infection and AAC, 25 of which were in pediatric or adult females (Agergaard and Larsen, 2015; Yesilbag et al., 2017; Harvey et al., 2021). Third, the first case of EBV-associated mixed gallbladder carcinoma was reported, which manifested a unique phenotype of lymphoepithelioma-like carcinoma and mucinous differentiated adenocarcinoma (Jain et al., 2021). Although the close correlation between EBV and GBC has been widely confirmed, more mechanistic research is needed to understand the pathogenesis and biological behavior of EBV-associated GBC.

HCV also appears to induce gallbladder lesions. HCV infection is a major cause of liver disease in elderly Chinese patients with chronic liver disease, which may be based on the HCV-induced increased risk of gallstones (Li and Gao, 2018). Moreover, a survey conducted in Taiwan with a high prevalence of HCV showed that HCV was associated with gallstone formation in men but not in women through univariate and multivariate analyses (Dai et al., 2013). Future studies can start from the perspective of the association and potential mechanisms between HCV and GBC carcinogenesis.

Human endogenous retrovirus (HERVs) are specifically activated in GBC and associated with cancer development. HERVs are a class of transposable elements formed from retroviral DNA segments integrated into the genome of germ cells millions of years ago, accounting for approximately 8% of the human genome (She et al., 2022). By single-cell RNA sequencing, study certified that there is aberrantly activated HERVs in GBC (Wang et al., 2022). HERVs are transcribed in a cell type-specific manner in GBC, as indicated by the increased HERVK11D-int, HERVE-int and HERVH-int in epithelial cells, the increased LTR58 in B cells, as well as the increased MLT1G in Monocytes. Mechanistically, dual luciferase reporter assays determine the enhancer activity of HERVs, which may cause changes in the expression of neighboring genes. Due to intratumoral cellular heterogeneity, multiple HERVs are present in GBC, which subsequently generate diverse complex biological effects. Interestingly, with the malignant transformation of gallbladder epithelial cells, the transcript levels of HERVH, the main HERV family expressed in those cells, gradually increased, suggesting that HERVH may be a potential biomarker for early diagnosis of GBC.

## 5 Fungi in GBC pathogenesis

Not only bacteria and viruses but also fungi are commonly latent in the gut, especially *C. albicans*, which is closely related to gallbladder disease (Spaulding et al., 2018). Fungi, may affect host health through secreted toxic substances or regulated host immune (Boutin et al., 2021).

Mycotoxins secreted by fungi, including Aflatoxin (AFT) and Ochratoxin A (OTA), appear to be high risk factors for GBC. A case-control study of GBC and gallstones patients determined that the AFT plasma exposure is associated with GBC (Koshiol et al., 2017). This study proposed that if AFT is the cause of GBC, this could contribute to the approximately 20% GBC in Shanghai, China and even higher rates in high-risk areas, suggesting that reducing AFT exposure may reduce the incidence of GBC. Interestingly, high level of red chili peppers (RCPs) consumption is identified as a risk factor for GBC in certain countries, such as Chile, Hungary, Bolivia and Peru (Nakadaira et al., 2009; Tsuchiya et al., 2011; Asai et al., 2012). Further studies confirmed that RCPs from these countries are contaminated with fungal-secreted AFT, which is subsequently ingested by human body and concentrated in gallbladder bile, leading to low-level but long-term AFT exposure and promoting GBC carcinogenesis. Another study found that mycotoxin OTA also contaminated RCPs from the countries such as Chile, Hungary, Bolivia and Peru, which was a risk factor for the development of GBC (Ikoma et al., 2015). On the one hand, compared to AFT, the RCPs in these countries are contaminated with higher concentrations of OTA. On the other hand, the average concentration of OTA in RCPs of Chili and Bolivia with high GBC morbidity is higher than that in Peru with moderate GBC morbidity. These research data all indicate that higher concentrations of OTA in RCPs have a greater association with GBC development compared to AFT. In addition, an ecological study was conducted in India, a country with high incidence of GBC and high consumption of RCPs (Ikoma et al., 2016). The results showed that there was no obvious correlation between mycotoxin concentration in red pepper and incidence rate of GBC in India. Therefore, more studies in human subjects rather than just RCPs are needed in the future to explore the association and underlying mechanisms between mycotoxins and GBCs.

*C. albicans* may coexist in the gut with the host when the host is healthy, but when intestinal barrier function is destroyed or human immune function is impaired, it invades the gallbladder and causes numerous gallbladder diseases (Yang et al., 2022b). Several studies found that *Candida* spp. can be cultured from bile or gallbladder tissues of patients with AAC (Hartmann and Schnabl, 2023), cholecystitis (Lai et al., 2005), cholangitis (Diebel et al., 1996), common bile duct obstruction (Gupta et al., 1985) cholelithiasis (Matyjas et al., 2017), gallbladder mass (Jajoo et al., 2012) and GBC (Yamamoto et al., 1994), which reflects the strong link between *Candida* spp. infection and gallbladder disease. Surprisingly, *C. albicans* can persist in gallbladders under antifungal therapy (Hsieh et al., 2017), which may benefit from *C. albicans* resistance to multiple antifungal agents in bile. However, the pathogenicity and specific mechanism of *Candida* spp. in GBC have not yet been recognized.

## 6 Conclusion

As one of the most complex, devastating and least-understood human pathologies, GBC progression is closely linked to changes in the composition and function of gut microbiota (Upadhyay, 2021). Although still controversial, research over the past 15 years has provided meaningful evidence into the relationships between gut microbiota and GBC, as well as their potential mechanisms of action. This review summarizes the existing evidence regarding the various gut microbiota and GBC progression, including the three major aspects of bacteria, viruses, and fungi. However, it is obvious that recent mechanistic studies mainly focus on bacteria, while other types of microorganisms, especially viruses and fungi, are almost exclusively addressed in partial case reports. Intestinal viruses and fungi play essential roles in fecal transplantation therapy through immunomodulation, thereby affecting human health (Lam et al., 2022). Therefore, in the future, more research on the mechanisms of GBC-associated gut microbiota should be performed, especially regarding the roles of viruses and fungi.

The majority of patients with GBC present with advanced and unresectable disease at the time of diagnosis due to poor prognosis, thus the identification of powerful and promising GBC-associated biomarkers is an imminent need. Based on IARC data, only 11 infectious pathogens (including 7 viruses, 3 flatworms and 1 bacterium) are formally recognized as grade 1 carcinogens for human cancers (Cullin et al., 2021). However, no unique carcinogenic microorganism has been identified that causes GBC, and the list has not been updated in the past decade (de Martel et al., 2020). GBC appears to be the result of the combined action of multiple mixed microorganisms that promote cancer progression but are not enough to cause cancer alone through a variety of mechanisms (virulence factors, metabolites, inflammation and ecological niche). Since there are still many potential microorganisms, as well as puzzling microbial mechanisms underlying the development of GBC, we need to conduct extensive research in the future to explore the relationship between gut microbiota and GBC, especially for known bacteria, viruses, and fungi. Despite many formidable challenges, a better understanding of the causality, role, and molecular mechanisms between commensal gut microbiota and GBC may offer a strong theoretical basis for improving the early diagnosis, prevention, treatment and prognosis of patients.

## Author contributions

SL: Conceptualization, Writing – original draft, Writing – review & editing. WL: Writing – review & editing. JC: Writing – review & editing. ML: Conceptualization, Formal Analysis, Investigation, Visualization, Writing – review & editing. YG: Conceptualization, Supervision, Validation, Visualization, Writing – review & editing. YL: Supervision, Validation, Visualization, Writing – review & editing. WW: Conceptualization, Data curation, Formal Analysis, Funding acquisition, Investigation, Methodology, Project administration, Resources, Supervision, Validation, Visualization, Writing – review & editing.
